# Dysregulated blood biomarkers in women with acute and chronic respiratory conditions due to air pollutant exposure: An exploratory systematic review

**DOI:** 10.7189/jogh-14-04207

**Published:** 2024-11-08

**Authors:** Ariana N Pritha, Tanisha N Medha, Andrea A Pasmay, Md Al Mamun, Farzana Afroze, Mohammod Jobayer Chisti

**Affiliations:** 1 Department of Neuroscience, Health Sciences Center, University of New Mexico School of Medicine, Albuquerque, USA; 2 Department of Internal Medicine, Health Sciences Center, University of New Mexico Comprehensive Cancer Center, Albuquerque, USA; 3 Library, International Centre for Diarrhoeal Disease Research, Bangladesh, Dhaka, Bangladesh; 4 Dhaka Hospital, Nutrition Research Division, International Centre for Diarrhoeal Disease Research, Bangladesh, Dhaka, Bangladesh

## Abstract

**Background:**

Air pollution exposure poses significant health risks for the general population, but particularly for women with acute and chronic respiratory conditions. Given the increasing global burden of air pollution-related illnesses, understanding these biomarkers is crucial for developing targeted interventions and improving respiratory health outcomes in vulnerable populations. In this systematic review, we aimed to determine potential dysregulated respiratory inflammatory blood biomarker candidates in adult female patients who experience varying levels and sources of inhaled pollutant exposure.

**Methods:**

We searched the Cochrane Library, PubMed, and Web of Science with nuanced search terms to retrieve articles published in English between 1 January 2000 and 12 June 2023, to ensure relevancy. We filtered our findings to generate a focussed narrative analysis and used the Risk of Bias In Non-randomized Studies-of Exposures (ROBINS-E) and Risk-of-bias VISualization (robVIS) tools to ensure the validity of the data and the quality of the conclusions being made.

**Results:**

We identified 916 articles from the databases used in our search, 16 of which met the criteria of our focussed narrative analysis. Among blood biomarkers, platelet-activating factor and eosinophilia could be used to assess the severity of asthma conditions, as a lack or reduction thereof indicates specific conditions. Pro-inflammatory cytokines require further validation, as some studies with a high risk of bias have reported conflicting results compared to more recent research on whether these markers are up-regulated or down-regulated. We found one study to be at a very high risk of bias, two had a high risk of bias, one had some concerns of confounding factors which may not have affected their results, and 12 studies had a low risk of bias.

**Conclusions:**

There were narrowed-down blood biomarker candidates that could be used in future research and avenues of research like generating specific microRNA sequences to test for prognostic/diagnostic tests.

**Registration:**

PROSPERO: 42023435721.

There is a significant risk of constant air pollution in Bangladesh which negatively impacts foetal development during pregnancy and infant development. Oxidative stressors, primarily via ozone exposure, can increase the risk of pneumonia and pregnancy complications [1]. Meanwhile, ozone exposure, a common component of air pollution, is known to worsen respiratory conditions in children, with breastfeeding playing a crucial role in reducing these harmful effects. The adverse impacts of ozone include increased incidences of asthma, bronchitis, and pneumonia among children, leading to increases in the number of hospitalisations and heightened respiratory distress.

Breastfeeding serves as a protective measure against the detrimental respiratory consequences induced by ozone exposure in children [2]. Breast milk is rich in antibodies, as well as immunological and bioactive components that strengthen the infant's immune system and support respiratory health [3]. It facilitates passive immunity by transferring maternal antibodies, which strengthens the infant's immune response to respiratory pathogens and reduces the severity of respiratory infections [4]. It also contains anti-inflammatory agents that alleviate respiratory symptoms and promote lung development [5], potentially reducing the inflammatory repercussions of ozone exposure on the respiratory system [6]. Furthermore, breastfeeding is associated with a lower risk of sudden infant death syndrome, a condition that can be worsened by respiratory infections and air pollution [7]. By promoting optimal lung function and respiratory health, it reduces susceptibility to respiratory complications caused by ozone exposure [8]. In regions with high air pollution levels, promoting and supporting breastfeeding practices is an essential public health intervention to protect children from ozone-induced respiratory problems [9,10]. Biomarkers detectable in maternal blood serum can be used to identify chronic ozone exposure and respiratory inflammation in both expecting mothers and those who have been breastfeeding for six months or longer during hospital visits, helping to assess foetal health [11].

Air pollution is one of the primary risk factors for mortality in Bangladesh [12], with current research suggesting that prenatal exposure to ozone in early and later stages of pregnancy can increase the risk of a deprived environment in the womb, negatively affecting child development [13]. This issue is particularly severe in lower socioeconomic countries like Bangladesh, where pneumonia is the leading cause of childhood death [14]. This leaves a need for epidemiological studies and basic science research to help reduce childhood mortality. Investigating biomarkers related to respiratory inflammation from air pollution and other early-stage pneumonia markers is particularly important for pregnant mothers and infants. Short-term exposure to ambient ozone has been shown to increase pneumonia-related hospitalisations and can become fatal for infants [15], however, there has been little research on the long-term effect of ozone exposure on the risk of developing pneumonia and related pregnancy complications.

Exposure to high levels of ozone has been linked to an increased risk of developing pneumonia [15–17]. Ozone is a highly reactive gas that can damage functional units of the lungs, often leading to compromised lung function [18]. When inhaled, it can react with lung tissue below the terminal bronchioles, the functional units of the lungs, leading to inflammation and other harmful effects, which in turn results in an increased risk of developing chemical pneumonia, especially in people who have pre-existing lung conditions or weakened immune systems [19]. An assessment factor for ozone exposure is often referred to as oxidative stress, which occurs when there is an imbalance between the production and accumulation of reactive oxygen species (ROS) in cells and tissues, and the capacity of the biological system to neutralise and remove these reactive substances.

Some other potential inflammatory blood biomarkers related to immune pathways include, but are not limited to, interleukins (IL), microRNAs, anti-inflammatory proteins, and many more. To discuss the mechanisms of a few candidates, the Clara cell protein 16 (CC16), for example, is a protein produced by the cells lining the airways in the lungs, known as Clara cells [20]. It plays a role in protecting the lungs from injury and inflammation [21]. IL-6, in turn, can also activate the nuclear factor kappa-light-chain-enhancer of activated B cells (NF-κβ) pathway, which promotes the expression of proinflammatory genes and antagonises the anti-inflammatory effects of glucocorticoids [22]. This can lead to glucocorticoid insensitivity, which can contribute to the development of respiratory diseases associated with ozone exposure [23]. Surfactant protein D (SP-D), meanwhile, is a protein that is produced in the lungs and is involved in innate immune defence. SP-D plays a role in the clearance of pathogens and debris from the lung and also modulates the inflammatory response [24]. Exposure to ozone has been shown to decrease the levels of SP-D in the lungs, which may impair the ability of the lungs to clear pathogens and debris [25], as well as to disrupt the function of SP-D, leading to a decrease in its ability to modulate the inflammatory response [26].

Exposure to air pollution during pregnancy has been linked to a range of adverse outcomes, including preterm birth, low birth weight, intrauterine growth retardation, and stillbirth [27–29]. Air pollution can also worsen existing health conditions, such as asthma or other respiratory problems, which can increase the risk of complications during pregnancy. The chronic exposure to high levels of ozone during pregnancy can have a range of negative effects on both the mother and the developing foetus [30]. In this sense, many recent studies have found a relationship between a lack of breastfeeding and incidences of respiratory and gastrointestinal illnesses [31–33]. Future clinical trials, however, should aim to identify and characterise inflammatory markers in females of child-bearing age within specific regions, which would enable comparative investigations into the respiratory outcomes of their current and future children. Yet there is a significant knowledge gap in this area, both in epidemiological and basic science research. Our review aims to provide a basis for further studies aiming to characterise prognostic features for women with health care issues. We investigated biomarkers related to air pollutant exposure and their association with adverse respiratory conditions to assess their potential clinical utility for future diagnostics.

## METHODS

### Study design

We performed a narrative systematic review to compile data for comparison, but did not perform any pooled analysis. Specifically, we narrowed down respiratory conditions with air pollution as a common aetiology and categorised the result tables based on the type of pollutant exposure. We analysed female adult patients in general as patterns among that demographic could be translatable in mothers after the postpartum stage.

We modified the population, intervention, comparison, and outcome (PICO) components as ‘population, ‘interest’, and ‘context’ to design the following question for our review: ‘What are potential dysregulated respiratory inflammatory blood biomarker candidates in adult female patients who experience levels of inhaled pollutant exposure causing deteriorating respiratory health?’

The primary outcomes were distinct primary blood inflammatory markers (such as ROS), including ozone, nitrogen oxide, sulfur dioxide, and others associated with pulmonary damage. The secondary outcomes were trends in markers that may be unique to the female population.

### Search criteria

We conducted this review by following the PRISMA guidelines. We selected the search criteria *a priori*, with key terms written in Boolean string search structure for possible synonyms of concepts relating to the objective, alongside MeSH were used where applicable.

In terms of specific keywords and terms, we noted that in respiratory human models, sputum collection is typically performed more frequently than blood draws. As a result, we included various synonyms and alternatives for blood (i.e. plasma and serum) to avoid limiting our analysis to studies that focus solely on sputum.

**Figure 1:**
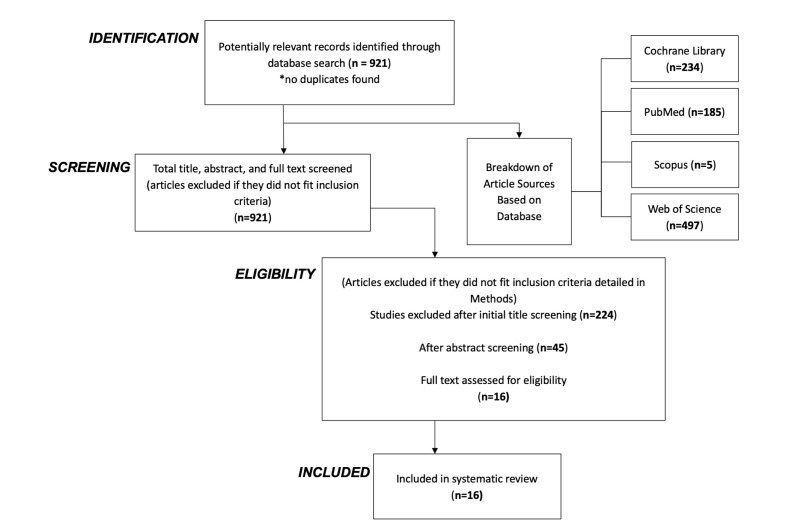
Modified PRISMA flow diagram for study selection.

The International Centre for Diarrhoeal Disease Research in Bangladesh head librarian (MAM) helped to screen and confirm the quality of database-specific search strategies to the initial search criteria to expand the search results as per PRISMA guideliens (**Figure 1**). An example of one search strategy follows:

(“blood” or “plasma” or “serum”) AND (“pulmonary” or “respiratory” or “aspiration” or “inhalation”) AND (“ozone” or “oxidative stress” or “air pollution” or “Free radical damage” or “Oxidative damage” or “Redox imbalance”) AND (“markers” or “biomarkers” or “molecular marker” or “signature molecule”) AND (“adult”) AND (“female”) AND (“human”).

We added additional filters as per the inclusion criteria when conducting an advanced search in each database.

### Database selection

We selected PubMed, Web of Science, Scopus, and Cochrane Library (including ClinicalTrials.gov, International Clinical Trials Registry Platform, and Embase), with language set as English only as an inclusion criterion.

We included both completed interventional (i.e. experimental and quasi-experimental trials) and observational studies with a timeframe between 1 January 2000 and 12 June 2023, in which the patient population was female adults aged 18 years and above. We also included studies and trials with pregnant women and studies with a roughly equal male-to-female ratio that showed no statistical significance in sex-dependent alterations in markers in order to broaden the range of candidate markers. We otherwise excluded review articles, case reports, case series, research on animal models, and studies that focussed solely on genetic respiratory conditions, cardiovascular effects, signs or symptoms, or any other factor causing respiratory conditions unrelated to air pollution.

### Screening process

Two researchers (ANP and FA) screened the title/abstracts, followed by the full texts of the retrieved references separately. A third researcher (MAM) helped resolve any discrepancies in the process, while a fourth researcher (MJC) helped ensure that no duplicate studies were include in the analysis.

### Data extraction

To ensure data comparability, we manually extracted the following characteristics of the included studies into a standardised extraction sheet: article reference, publication year, key details regarding their intervention (if any), study design, type of pollutant exposure, respiratory condition of focus, co-existing conditions (if any), number of female participants, types of analysed markers, and female participants’ characteristics (i.e. respiratory illness of focus, patient outcome if reported, etc.). All co-authors verified the extracted data to ensure accuracy.

### Quality assessment of included studies through risk of bias analyses

We used the Risk of Bias In Non-randomized Studies – of Exposures (ROBINS-E) tool as it effectively assesses the risk of bias in observational studies of exposures [34]. It has seven and is the only tool available to assess bias in exposure studies. We therefore preferred it over the ROBINS-of Interventions (ROBINS-I) tool because the ROBINS-E domains included criteria matching the ROBINS-I tool that would allow for more accurate risk of bias assessment in combined exposure and intervention studies, but the reverse was not true, as ROBINS-I focusses on the risk of bias in observational intervention studies. Two independent reviewers conducted the risk of bias analysis by carefully considering each domain (Appendix S2 in the **Online Supplementary Document**). To ensure consistency and accuracy, we conducted a thorough discussion among the reviewers, through which we reached a consensus on the interpretation of the scale, leading to a more cohesive evaluation process.

**Figure 2:**
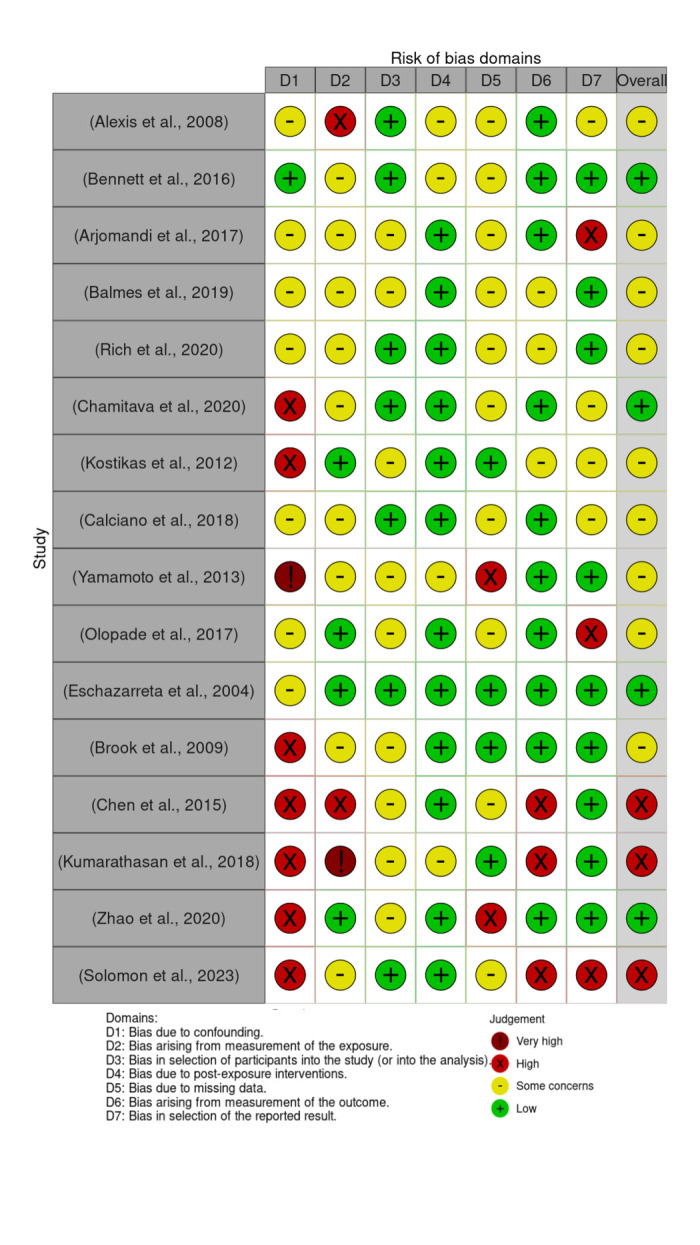
robVIS tool consolidated results displaying risk of bias summary results generated from ROBINS-E tool results [35].

After we manually filled out the ROBINS-E survey for each article through a series of yes and no questions (including to what extent) and exported that data into another Excel file formatted by the robVIS tool, we uploaded that file to the robVIS tool to compare strengths and weaknesses comprehensively between all articles (**Figure 2**).

### Data synthesis and analysis

Since this is a comparative narrative systematic review, we used connections between and outside the included articles to rationalise the mechanisms behind the selected inflammatory markers that are more or less effective than other candidates. The decision to adopt a narrative review approach was driven by the considerable heterogeneity observed among the included studies, stemming from variations in study designs, outcomes, and data quality. This approach facilitated a qualitative synthesis of the varied findings, offering insights into key themes, trends, and areas of agreement or disagreement across the literature. While we acknowledge its limitations, the narrative synthesis provided a comprehensive overview of the evidence landscape and served as a foundation for guiding future research directions in the field.

## RESULTS

We retrieved 234 articles from the Cochrane Library, 185 from PubMed, five from Scopus, and 497 from the Web of Science. After the title/abstract and full-text screening, we included two articles from the Cochrane Library, 12 from PubMed, and two from the Web of Science. There were no overlapping publications, but we did find different publications from the same clinical trial (those were not separated as different markers were reported, or each publication investigated a different research aspect). Many entries in databases were for ongoing clinical trials, those that had not yet started recruiting, and those that did not publish their data despite being completed.

Overall, we found that there were various biomarkers dysregulated across multiple studies (**Table 1****,** **Table 2****,** **Table 3**). Many inflammatory markers were not studied in these samples, leaving room for further research.

**Table 1:** Conditions from ozone exposure/reactive oxygen species*

Authors, year (reference)	Pollutant exposure	Type of study	Respiratory condition of focus	Co-existing conditions	Number of subjects	Types of analysed markers and results
Alexis et al., 2008 [36]	Ozone	Double-blind, placebo-controlled, randomised, three-period crossover study	O_3_-induced lung function decline	N/A	8 female (9 male)	CC16 levels: statistically significantly increased pre-vs post-O_3_ exposure.
						SP-D levels: not statistically significantly altered pre-vs post-O_3_ exposure.
Bennet et al., 2016 [37]	Ozone	Randomised controlled exposure study	O_3_-induced lung function decline	Obese	19 females	IL-6 levels: increased in healthy subjects’ pre-vs post-O_3_ exposure as well as in obese subjects but to a greater degree.
						FP significantly reduced the percent of neutrophils in sputum by 18% and 35% for 0.5 mg and 2 mg FP, respectively.
						FP also significantly reduced the total number of neutrophils per milliliter of sputum by 14% and 43% for 0.5 mg and 2 mg FP, respectively.
Arjomandi et al., 2018 [38]	Ozone	Randomised crossover-controlled exposure study, MOSES study	O_3_-induced lung function decline	Geriatric population; exercise intervention	52 females (35 males)	CC16 levels: four hours post-exposure, significantly increased pre-vs post-O_3_ exposure dependent on the concentration.
Balmes et al., 2019 [8]	Ozone	Randomised double-blind crossover study, MOSES study†	O_3_-induced lung function decline	Geriatric population; exercise intervention	52 females (35 males)	CRP, IL-6, 8-isoprostane, or P-selectin: no change.
						Nitrotyrosine: decreased after 120 ppb ozone.
Rich et al., 2020 [39]	Ozone	Randomised double-blind crossover study, MOSES study†	O_3_-induced lung function decline	Geriatric population; exercise intervention	52 females (35 males)	After extending the time for pre-collection and standardising for ambient air pollution, results changed.
						Increasing ambient NO_2_, CO, and PES altered pulmonary function responses in a concentration-dependent manner which means previous MOSES studies are accurately measuring markers’ relationships.
Chamitava et al., 2020 [40]	ROS	Observational study	COPD, chronic bronchitis, and asthma	Some smokers	Current asthma: 212 females. Past asthma: 84 females. Chronic bronchitis: 50 females. Controls: 2270 females	Chronic bronchitis subjects showed a higher rate of expression of oxidative stress biomarkers compared to asthmatic subjects.
s						Asthmatics: blood leukocytes, basophils, and eosinophils heightened with ozone exposure.
						Chronic bronchitis: glutathione and lymphocytes more heightened than normal.

C16 – club cell secretory protein 16, CO – carbon monoxide, COPD – chronic obstructive pulmonary disorder, CRP: C-reactive protein, FP – fluticasone propionate, IL-6 – interleukin 6, MOSES – Multicenter Ozone Study in oldEr Subjects, NO_2_ – nitrogen dioxide, O_3_ – ozone

PES – potential environmental susceptibility, ppb – parts per billion, ROS – reactive oxygen species, SP-D – surfactant protein D

*Potential ozone-related inflammation candidates: CC16, nitrotyrosine, and IL-16 dysregulated.

†Same trial as above, different marker analysis.

**Table 2:** Conditions from smoke from cigarettes/diesel exhaust/gas stove exhaust

Authors, year (reference)	Pollutant exposure	Type of Study	Respiratory condition of focus	Co-existing conditions	Number of subjects	Types of analysed serum markers and results
Kostikas et al., 2013 [41]	Second hand smoke exposure	Randomised controlled crossover trial	Range of inhibited lung capacities	N/A	9 females (9 males)	Cotinine: increase in level observed immediately post-O_3_ exposure and remained increased until the end of the trial.
						Lung function changes in FEV1 (significant reduction (F = 38.416, *P* < 0.001)) and FEF25-75 (significant reduction (F = 10.633, *P* < 0.001))
						FEV1/FVC ratio: significant reduction (*P *< 0.001)
						No significant changes observed in FVC (F = 0.270, *P* = 0.946) or PEF (F = 1.274, *P* = 0.300)
Calciano et al., 2018 [42]	Oxidative stress (cause from smoking or natural environment)	Population-based, multicentre, (multi)case-control study	Asthma	Smokers	140 female cases of asthma (total 287 cases)	Eosinophil, basophil, and lymphocyte levels: positively associated with increased risk of higher severity of respiratory symptom when paired with shortness of breath at rest as a sign.
Yamamoto et al., 2013 [43]	DE	Double-blinded, randomised crossover study	Asthma	N/A	13 participants (sex ratio unspecified but no significance between sexes)	Four microRNA candidates – hsa-miR-144, hsa-miR-30e, hsa-miR-21, hsa-miR-215.
						hsa-miR-144: increased levels post-DE exposure.
						hsa-miR-21: marginally significant results which were not discussed.
						Other microRNAs were not significant.
Olopade et al., 2017 [44]	Oxidative stress (stove kerosene vs ethanol vs firewood), ambient air: 72-h mean PM_2.5_	Randomised controlled trial	Lung function (cough, phlegm, wheeze, chest tightness, etc.)	Pregnant, but none with possibility of high-risk pregnancy	324 females	TNF-α: improved levels in households that used firewood prior to trial enrollment and shifted to ethanol, thus, cleaner fuels reduced inflammation.
						IL-8: increased at a lower rate among the ethanol vs kerosene stove users and the ethanol group when compared with kerosene users at baseline and the control group.

CRP – C-reactive protein, DE – diesel exhaust, FEF25–75 – forced expiratory flow at 25% to 75% of the pulmonary volume, FEV1 – forced expiratory volume in 1 second, FVC – forced vital capacity, hsa-miR – human microRNA, IL-8 – Interleukin 8, MOSES – Multicenter Ozone Study in oldEr Subjects, O_3_ – ozone, PEF – peak expiratory flow, PM2.5 – particulate matter with a diameter of less than 2.5 micrometers, ROS – reactive oxygen species, TNF-α – tumor necrosis factor-alpha

**Table 3:** Conditions from ambient air pollution/oxidative stress (no co-existing conditions reported)

Reference	Pollutant exposure	Type of study	Respiratory condition of focus	Number of subjects	Types of analysed markers and results
Echazarreta et al., 2005 [45]	Oxidative stress	Randomised, double-blinded, L-PAF-controlled, crossover study	Mild asthma	6 females (6 males)	PAF: no increase in systemic markers of oxidative stress in response to PAF as normally seen in acute asthma. L-PAF: no significant results. From PAF challenge, peripheral blood neutrophils fell at 5 min (*P* < 0.01) followed by rebound neutrophilia at 15 and 45 min (*P* < 0.001 each). Rrs (to 5.5 cm H2OL^−1^s^−1^ (SD = 0.4)) (*P* < 0.005) and AaPo_2_ (to 27.1 mmHg (SD = 2.8)) increased whereas Pao_2_ decreased (to 76.2 mmHg (SD = 2.1)) (*P* < 0.001 each) at 5 min. Heart rate increased (from 70 min (SD = 1.7) to 78 min (SD = 2.2) − 1) (*P* < 0.005) at 5 min.uLTE4 elimination increased markedly (to 2144 pgmg (SD = 845) − 1) (*P* < 0.02) at 120 min. From L-PAF challenge, there was a small increase in AaPo_2_ at 5 and 15 min (*P* < 0.01 each). There was also a mild decrease in Pao_2_ at 15 min (*P* < 0.01).
Brook et al., 2009 [46]	Air pollution	Randomised, double-blind study	Respiratory/vascular health	15 females (16 males)	ET-1, CRP, and cytokines: no significant differential change.
Chen et al., 2015 [47]	Outdoor particulate matter: PM_2.5_ were 103 μg/m^3^. Indoor (air purified) PM_2.5_ concentration: 96.2 μg/m^3^	Randomised, double-blind crossover study	Acute airway inflammatsion	25 females (10 males)	Serum biomarkers – CRP, fibrinogen, P-selectin, MCP-1, IL-1β, TNF-α, IL-6, MPO, sCD40L, PAI-1, t-PA, D-dimer,
					Improvements in all inflammatory markers pre- vs post-air filtration except for CRP.
Kumarathasan et al., 2018 [48]	Environmental air pollution exposure	Randomised crossover study	Airway inflammation	52 (male and female ratio not specified; no sex-based significance)	IL-1β, IL-2, IL-6: up-regulated with no mask on (filtered vs not filtered air).
Zhao et al., 2020 [49]	PM2.5 pollution waves	Randomised crossover trial	Respiratory inflammation	13 females (16 males)	Increased ox-LDL and 8-isoPGF2α levels with pollution exposure, other markers (MDA, GPx1, EC-SOD) not significant.
Solomon et al., 2023 [50]	Natural aetiology (can be environment, allergic response to environment, etc.)	Institution-based cross-sectional study (random sampling)	Asthma	164 females (127 males)	Eosinophilia: lacking levels associated with low type-2 asthma phenotype.

8-isoPGF2α – 8-iso-ptostaglandin F2-alpha, AaPO_2_ – alveolar-arterial oxygen gradient, CRP – C-reactive protein, EC-SOD – extracellular superoxide dismutase, ET-1 – endothelin-1, GPx1 – glutathione peroxidase 1, IL-1β, IL-2, IL-6 – interleukin 1-beta, 2, and 6, L-PAF – lyso-platelet-activating factor, MCP-1 – monocyte chemoattractant protein-1, MDA – malondialdehyde, a marker of oxidative stress, MPO – myeloperoxidase, PAF – platelet-activating factor, PAI-1 – plasminogen activator inhibitor-1, PaO_2_ – partial pressure of oxygen in arterial blood, PM2.5 – particulate matter with a diameter of less than 2.5 micrometers, Rrs – respiratory system resistance, sCD40L – soluble CD40 ligand, SD – standard deviation,

TNF-α – tumor necrosis factor-alpha, t-PA – tissue plasminogen activator, uLTE4 – urinary leukotriene E4, x-LDL – oxidized low-density lipoprotein

### Study characteristics

The included studies mostly used epidemiological cohort analysis and blood serum validation for molecular work. Participants were predominantly women, with a few studies focussing on both men and women. Interventions were environmental or induced and primarily an exposure to air pollution. The outcomes varied across studies but were similar for the same marker tested (**Figure 1**).

Ozone exposure can lead to oxidative stress and inflammation in the lungs, resulting in damage to the lower airway lining and decreased production of CC16 [51]. Alexis and colleagues report that this marker is increased which indicates that respiratory inflammation is being detected [36].

Nitrotyrosine is a marker of inflammation and nitric oxide production, and it was also reported to increase based on concentration but decreased past 120 ppb of ozone which may indicate some sort of mechanism to regulate tyrosine oxidation.

Bennett and colleagues reported IL-6 to be elevated [37], whereas Balmes and colleagues saw no change [8]. This is what leads to the risk of bias assessments to believe there is no consistency in the environment of subjects, so the studies are not comparable.

Balmes et. al found no change in CRP and SP-D, indicating that systemic inflammation may not affect that immunological pathway [8]. They also observed no change in 8-isoprostane [8], a marker of oxidative stress and lipid peroxidation, specifically related to the peroxidation of arachidonic acid [52]. This marker is produced during oxidative damage to cell membranes and tissues [53]. Ozone exposure can induce oxidative stress in the body, leading to the formation of ROS and subsequent lipid peroxidation [54]. 8-isoprostane is considered a reliable biomarker for assessing oxidative damage associated with ozone-induced inflammation [55]. This indicates that there was variability in the concentration of ozone exposure in this study by Balmes et al. [8] compared to other studies (**Table 1**).

Cotinine is a marker for nicotine exposure and a persistent induced result, as seen by Kostikas and colleagues [41]. Chamitava et al. [40] and Calciano et al. [42] observed an increase in this marker among asthmatic subjects, the former from ozone exposure and the latter from a smoking environment. While these markers may not be ideal for distinguishing between different pollutant exposures, they can still be useful in indicating whether airway inflammation is present in asthma patients and to what extent (**Table 2**). Eosinophils play a role in the immune response to parasitic infections and allergic reactions [56,57]. They are also involved in inflammation associated with respiratory diseases such as asthma.

Eosinophil-derived neurotoxin (EDN) is a protein that is released by eosinophils, a type of white blood cell involved in the immune response [58]. Studies have shown that exposure to ozone can lead to an increase in blood levels of EDN, indicating activation of eosinophils and potential involvement in ozone-related respiratory inflammation [59,60]. High levels of blood eosinophils have been associated with increased asthma exacerbations, suggesting that monitoring eosinophil levels may be important in predicting and preventing exacerbations in individuals exposed to ozone [61]. Higher EDN levels have also been associated with increased severity of asthma and other respiratory conditions [62,63].

Combustion from diesel exhaust often produces ozone as a byproduct. IL-8 is a chemokine that is produced in response to inflammation and can be used as a biomarker for the detection of ozone-induced inflammation [64]. Studies have shown that IL-8 levels are increased in the respiratory system of individuals exposed to oxidative stress and that blocking IL-8 signalling can reduce ozone-induced inflammation and airway hyperresponsiveness [51,65–67]. Olopade and colleagues demonstrated that there is a relationship between the type of diesel exhaust exposure and the magnitude of increased levels [44]. Other studies have shown that exposure to ozone can lead to increased levels of IL-8 in the respiratory system, as well as in blood and other biological fluids [66,68,69].

In response to ozone exposure, ROS are generated in the lung cells, which trigger NF-κβ and activating protein-1 [70,71]. These transcription factors then bind to the promoter regions of genes encoding pro-inflammatory cytokines, including tumour necrosis factor (TNF-α), and stimulate their expression. TNF-α, in turn, can activate other immune cells, such as neutrophils and macrophages, which can further exacerbate the inflammatory response and tissue damage [72,73]. TNF-α can also promote the migration of immune cells into the lung tissue, further exacerbating the tissue damage [74]. Additionally, TNF-α can induce the expression of adhesion molecules on the endothelial cells that line the blood vessels in the lungs, promoting the adhesion and migration of immune cells into the lung tissue [75].

Platelet-activating factor (PAF) could be used as a potential marker to differentiate between mild and acute asthma, which may help reduce the use of steroids in patients with mild asthma, allowing for more conservative treatment options (**Table 3**). In response to this oxidative stress from ozone, cells like macrophages and epithelial cells release IL-1β as part of the inflammatory response [76], IL-1β acts as a signalling molecule, recruiting and activating other immune cells to the site of inflammation [77]. It promotes the expression of adhesion molecules on endothelial cells, allowing immune cells to adhere to the blood vessel walls and migrate into the affected tissues [69]. IL-1β also stimulates the production of other pro-inflammatory cytokines and mediators, amplifying the inflammatory response [78].

8-isoprostane is a marker of oxidative stress and lipid peroxidation, specifically related to the peroxidation of arachidonic acid [52]. It is produced during oxidative damage to cell membranes and tissues [53]. Ozone exposure can induce oxidative stress in the body, leading to the formation of ROS and subsequent lipid peroxidation [54]. Elevated levels of 8-isoprostane in biological samples, such as urine or exhaled breath condensate, indicate increased oxidative stress and lipid peroxidation caused by ozone exposure [79]. For this reason, it is considered a reliable biomarker for assessing oxidative damage associated with ozone-induced inflammation [55].

## DISCUSSION

In this review, we identified potential candidates for future clinical trials aimed at distinguishing the severity of respiratory conditions like asthma while providing significant insights into ozone-related inflammation markers. Specifically, CC16, nitrotyrosine, and IL-16 exhibited dysregulation post-ozone exposure, indicating oxidative stress and inflammation in the lungs. While Alexis and colleagues reported increased CC16 levels, indicating detected respiratory inflammation [36], nitrotyrosine levels notably increased based on the concentration, although decreasing past 120 ppb of ozone, potentially suggesting a regulatory mechanism for tyrosine oxidation. However, inconsistency in IL-6 levels between Bennett et al. and Balmes et al. underscores the challenge of comparability among studies due to environmental variations among subjects [37,53].

Contrarily, SP-D, CRP, and 8-isoprostane exhibited no ozone-related dysregulation, indicating potential unaffected pathways. While CRP and SP-D showed no change, indicating negligible systemic inflammation impact, Balmes et al. also found no alteration in 8-isoprostane, questioning the variability in ozone concentration within the study [53].

Moreover, while cotinine persisted as a marker for nicotine exposure, varying results from Kostikas et al., Chamitava et al. [40], Kostikas et al. [41], and Calciano et al. [42] hint at its limited candidacy in differentiating between pollutant exposures [56,57]. Eosinophils and EDN revealed a potential link to ozone-related respiratory inflammation, with increased blood levels suggesting activation and involvement in inflammatory processes. IL-8 and TNF-α also demonstrated potential as biomarkers, reflecting ozone-induced inflammation severity and tissue damage. PAF emerges as a promising marker for differentiating mild and acute asthma, potentially reducing steroid use in patients with milder conditions. Finally, IL-1β and 8-isoprostane underscore the intricate interplay between oxidative stress, lipid peroxidation, and ozone-induced inflammation, suggesting their utility as reliable biomarkers for assessing inflammatory responses.

A few candidates emerged that could be used for future clinical trials to distinguish between the severity of respiratory conditions like asthma, but sample sizes for the variety of potential biomarkers in the included studies were small. CC16 was significantly increased after ozone exposure, as were IL-6 levels. Contrary to newer literature claiming that a decrease in CC16 levels has been suggested as a potential biomarker for ozone-induced lung injury [80], Alexis and colleagues report an increased level of CC16 which may result from too high of an ozone dose used [36]. There was a discrepancy within the CC16 research for the Multicenter Ozone Study in oldEr Subjects (MOSES) study, especially since the latest research indicated an opposite trend [81,82]. However, this may be because the classification of acute vs persistent pollutant exposure changed in a later phase of the trial which was published separately. This caused the risk of bias to be higher in those studies as later statistical corrections tried to reduce the confounding factors that may be involved in their analyses and data collection processes. Specifically, the studies by Chen et al. [47], Kumarathasan et al. [48], Zhao et al. [49], and Solomon et al. [60], had higher risks of bias (Figure S2 in the **Online Supplementary Document**).

Elevated levels of 8-isoprostane in biological samples, such as urine or exhaled breath condensate, indicate increased oxidative stress and lipid peroxidation caused by ozone exposure [79]. However, Balmes and colleagues found no change in levels of pre- vs post-ozone exposure [8]. Based on the identified limitations we observed in this field, we suggest that future studies should assess sex differences more in-depth by raising the power of studies. In sum, it might be beneficial to characterise all inflammatory cytokines relevant to respiratory inflammation in a controlled environment as different regions have varying levels of pollution, so it is not possible to compare ‘controls’ in studies [8]. 

In the context of the included studies, blood serum inflammatory markers are examined as potential indicators of dysregulation caused by air pollutant exposure in expecting mothers. While there is a substantial analysis of these markers in females, limited data are available for males. Notable markers assessed in females include CC16, SP-D, IL-6, 8-isoprostane, P-selectin, CRP, nitrotyrosine, blood leukocytes, basophils, eosinophils, glutathione, lymphocytes, cotinine, hsa-miR-144, hsa-miR-30e, hsa-miR-21, hsa-miR-215, TNF-α, PAF, L-PAF, ET-1, IL-1β, MPO, sCD40L, PAI-1, t-PA, D-dimer, IL-2, IL-8, ox-LDL, MDA, 8-isoPGF2α, GPx1, and EC-SOD. However, it is challenging to determine the duration of air pollutant exposure in patients, whether it is short-term or chronic, especially in studies that involve self-reporting and a lack of ‘control’ human samples that were removed from pollution to mark as baseline.

The secondary outcome analysis yielded inconclusive results, emphasising the need for better characterisation of female patient outcomes during pregnancy in comparison to male population data. Detecting chronic ozone exposure and respiratory infection parameters in expecting mothers can be vital for identifying high-risk individuals based on their geographical location, while exploring the connection between maternal immunity and the severity of pneumonia in breastfed infants can inform targeted interventions for maternal and foetal health improvement.

This research also holds significance for public health initiatives and future investigations. Monitoring chronic ozone exposure and respiratory infection features in expecting mothers can provide insights into the long-term effects of air pollution on maternal and foetal health and can guide the development of policies and interventions to mitigate exposure and enhance health outcomes. While many inflammatory markers have been assessed in the included studies, there is potential to refine the selection for a more effective evaluation of respiratory distress. Moreover, the wide range of settings in which the studies have been conducted likely had varying levels of pollution and unique environmental factors, reflecting real-world scenarios faced by populations globally. By considering these diverse contexts, the findings gain broader applicability, enhancing their relevance to populations experiencing varying degrees of ozone exposure and respiratory conditions. Thus, while quantitative comparisons may be limited, the robustness of the findings stemming from our analysis of currently available literature lies in their potential to inform interventions and policies across diverse geographical and environmental settings, strengthening their generalisability and impact on public health outcomes. There is limited research on serum markers in expecting mothers, with only one eligible article reporting data exclusively on pregnant women. To address this gap, further clinical trials and longitudinal studies in this population are needed, along with more standardised immune marker quantifications.

## CONCLUSION

Inflammatory markers are an accurate method of assessing systemic inflammation which can lead to lower airway infection. However, for large prognostic studies, baseline ranges need to be established in populations from the same race or region without exposure to city-level air pollution to ensure accurate diagnoses. Eosinophilia and PAF may be useful markers for assessing asthma severity, as their absence can indicate different levels of the condition. Our findings suggest that there is a necessity in large-sample randomised control clinical trials that can investigate a plethora of markers for analyses. Investigating potential microRNA sequences can also be the next step in this field.

## Additional material


Online Supplementary Document
