# Supplementary material for: Dysregulated blood biomarkers in women with acute and chronic respiratory conditions due to air pollutant exposure: An exploratory systematic review
Source: J Glob Health. 2024 Nov 8;14:04207. doi: 10.7189/jogh-14-04207 (PMC11544524; doi:10.7189/jogh-14-04207)

Appendix S1:

Table S1: Table summarizing results of included studies.

**Article Reference**	**Country of Origin of Study**	**Number of Patients Included in Completed Study**	**Key Findings from Article**
Eschazarreta et al.	Spain	12	There was no significant increase in systemic oxidative stress, as indicated by levels of malondialdehyde (MDA) and 8-isoprostane, in mild asthmatics after PAF challenge.
Alexis et al.	USA	17	FP treatment reduced percentage of neutrophils in sputum, with increased reduction of neutrophils in higher dosages of FP. FP did not significantly effect on O3-induced changes in lung function, IOS end points, MPO or total protein levels in sputum, or MPO/total protein ratio. In the immune system, FP increased the expression of CD11b, mCD14, CD64, CD16, HLA-DR, and CD86 on sputum monocytes. FP also increased production of Clara cell protein 16 (CCP16) and decreased production of myeloperoxidase (MPO).
Brook et al.	Canada/USA	81	Air pollution exposure can increase diastolic blood pressure, impair endothelial function, and increase tumor necrosis factor α. Air pollution exposure has a more pronounced effect on diastolic blood pressure, endothelial function, and inflammation, than on heart rate variability and brachial artery reactivity. Air pollution can indirectly affect the cardiovascular system by increasing inflammation and oxidative stress. Air pollution particles can directly damage the vascular endothelium, the lining of the blood vessels.
Kostikas et al.	Greece	18	Secondhand smoke exposure can acutely induce airway acidification and oxidative stress in healthy non-smokers. The lung function was unaffected from changes in EBC pH and H2O2 levels.
Yamamoto et al.	Canada	13	Diesel exhaust exposure induced significant changes in miRNA expression in PBMCs of healthy volunteers. NAC decreased the expression of miR-21, miR-30e, miR-215, and miR-144, all of which are involved in regulating inflammation and oxidative stress. Changes in miRNA expression were associated with increased levels of inflammatory markers.
Chen et al.	China	35	Air purifiers can reduce indoor levels of particulate matter of outdoor origin. Air purifiers can reduce lung inflammation, improve lung function, and lower blood pressure and heart rate.

Bennett et al.	USA	38	Obese women had greater decreases in forced vital capacity (FVC) and forced expiratory volume in 1 second (FEV1) after ozone exposure than non-obese women. Obese women also had greater increases in airway resistance and reactance after ozone exposure than non-obese women. Obese women had higher levels of sputum neutrophils and myeloperoxidase (MPO) after ozone exposure than non-obese women.
Arjomandi et al.	USA	87	Ozone exposure caused decreases in forced vital capacity (FVC) and forced expiratory volume in 1 second (FEV1) in older adults.<br>Ozone exposure also caused increases in airway resistance and reactance in older adults. The effects of ozone exposure were more pronounced in older adults with underlying lung disease.
Olopade et al.	Nigeria	324*	The intervention group (using clean stoves) had significantly lower levels of tumor necrosis factor alpha (TNF-α) and interleukin-6 (IL-6) than the control group (using traditional stoves) at 12 weeks postpartum. The intervention group also had significantly higher levels of the anti-inflammatory cytokine interleukin-10 (IL-10) than the control group at 12 weeks postpartum.
Calciano et al.	Italy	287	Eosinophils in sputum were positively associated with respiratory symptoms and lung function. FeNO (fractional exhaled nitric oxide) was positively associated with respiratory symptoms and lung function. Periostin in serum was negatively associated with respiratory symptoms and lung function. The combination of eosinophils, FeNO, and periostin gives a good prediction of respiratory symptoms and lung function.
Kumarathasan et al.	Canada	52	Air pollution exposure can increase diastolic blood pressure, impair endothelial function, increase tumor necrosis factor α, increase the expression of pro-inflammatory genes and increase the production of inflammatory cytokines.
Balmes et al.	US	1,062	Ozone exposure increased the levels of inflammatory markers (tumor necrosis factor-alpha (TNF-α) and interleukin-6 (IL-6)), oxidative stress markers (malondialdehyde (MDA) and 8-isoprostane) and impaired endothelial function (measured by flow-mediated dilatation). Ozone exposure increased the levels of pro-thrombotic markers (tissue factor (TF) and fibrinogen).
Rich et al.	US	87	Personal measures of ozone and air pollutants, such as NO2 and O3, can modify the effects of controlled

			O3 exposure on pulmonary function and reduce lung function.
Chamitava et al.	Italy	1878	Oxidative stress and inflammation are important pathophysiological mechanisms in chronic airway diseases, such as asthma and chronic obstructive pulmonary disease (COPD).<br>Biomarkers of oxidative stress and inflammation can be used to assess the severity of these diseases and to monitor the response to treatment. It is likely that biomarkers of oxidative stress and inflammation may be useful for predicting the risk of developing chronic airway diseases.
Zhao et al.	China	29	Short-term exposure to high levels of ambient PM2.5 was linked with increased respiratory symptoms, reduced lung function, and altered heart rate variability in young healthy adults. Effects of PM2.5 exposure were elevated in participants who were exposed to higher levels of PM2.5 and participants who had a history of asthma or allergies.
Solomon et al.	Ethiopia	291	Eosinophilia is common among asthmatic patients, especially those with severe asthma. This condition is linked to increased airway inflammation, airway hyperresponsiveness, and worse asthma control. In the study, patients with eosinophilia had worse asthma control than those without eosinophilia.

Appendix S2:
Summary of each reviewer's Risk of Bias assessments prior to consolidation.

Figure S1: Reviewer 1 Raw Data for Risk of Bias

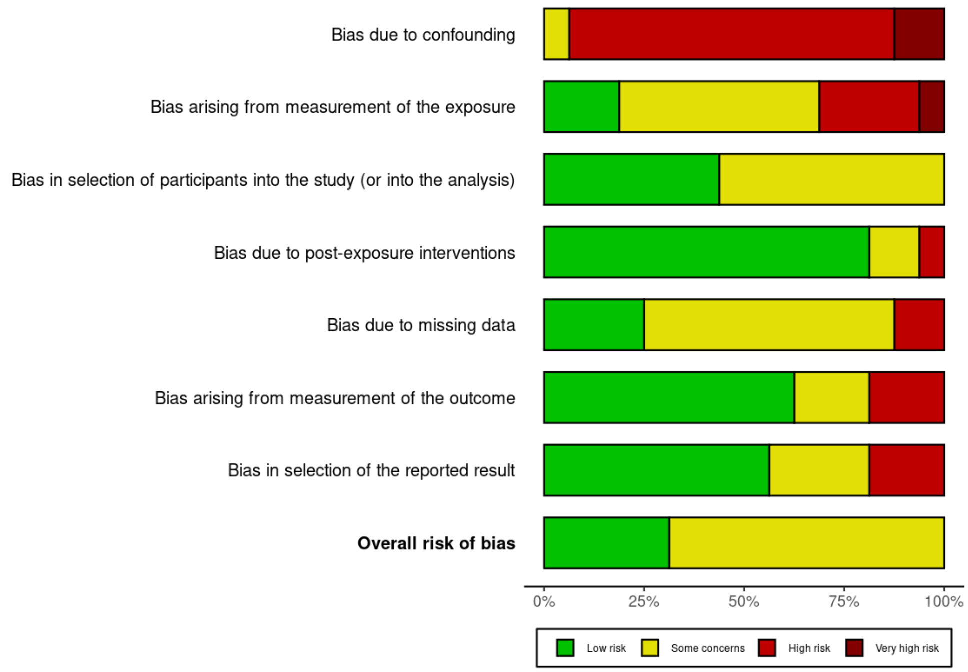

Figure S2: Reviewer 2 Raw Data for Risk of Bias

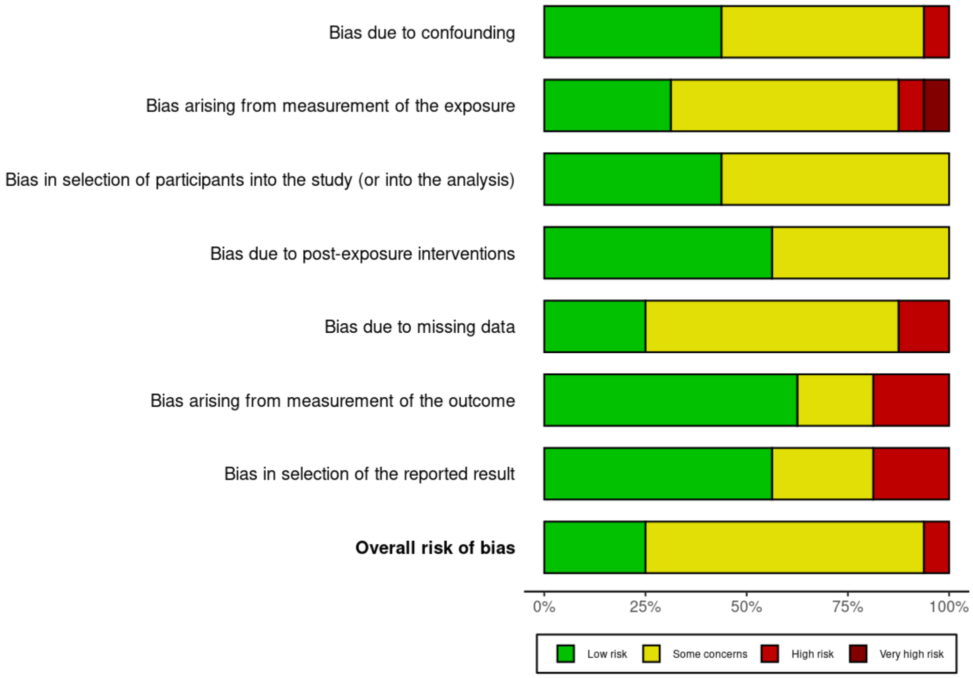

Supplement: Online Supplementary Document [file jogh-14-04207-s001.pdf]
